# Predation cues induce predator specific changes in olfactory neurons encoding defensive responses in agile frog tadpoles

**DOI:** 10.1371/journal.pone.0302728

**Published:** 2024-05-02

**Authors:** Andrea Gazzola, Daniela Ratto, Fabio Perrucci, Alessandra Occhinegro, Roberta Leone, Francesca Giammello, Alessandro Balestrieri, Daniele Pellitteri-Rosa, Paola Rossi, Federico Brandalise

**Affiliations:** 1 Department of Earth and Environmental Sciences, University of Pavia, Pavia, Italy; 2 Department of Biology and Biotechnology “L. Spallanzani”, University of Pavia, Pavia, Italy; 3 Department of Biomedical and Neuromotor Sciences, University of Bologna, Bologna, Italy; 4 Department of Environmental Sciences and Policy, University of Milan, Milano, Italy; 5 Department of Biosciences, University of Milan, Milano, Italy; Uppsala Universitet, SWEDEN

## Abstract

Although behavioural defensive responses have been recorded several times in both laboratory and natural habitats, their neural mechanisms have seldom been investigated. To explore how chemical, water-borne cues are conveyed to the forebrain and instruct behavioural responses in anuran larvae, we conditioned newly hatched agile frog tadpoles using predator olfactory cues, specifically either native odonate larvae or alien crayfish kairomones. We expected chronic treatments to influence the basal neuronal activity of the tadpoles’ mitral cells and alter their sensory neuronal connections, thereby impacting information processing. Subsequently, these neurons were acutely perfused, and their responses were compared with the defensive behaviour of tadpoles previously conditioned and exposed to the same cues. Tadpoles conditioned with odonate cues differed in both passive and active cell properties compared to those exposed to water (controls) or crayfish cues. The observed upregulation of membrane conductance and increase in both the number of active synapses and receptor density at the postsynaptic site are believed to have enhanced their responsiveness to external stimuli. Odonate cues also affected the resting membrane potential and firing rate of mitral cells during electrophysiological patch-clamp recordings, suggesting a rearrangement of the repertoire of voltage-dependent conductances expressed in cell membranes. These recorded neural changes may modulate the induction of an action potential and transmission of information. Furthermore, the recording of neural activity indicated that the lack of defensive responses towards non-native predators is due to the non-recognition of their olfactory cues.

## Introduction

Predator-prey interactions are a major selective force, shaping individuals’ morphology, life history, and behaviour, as well as population dynamics and the diversity of communities [1, 2]. Predation events are hypothesized to follow a series of steps: detection, attack, capture, and ingestion [3]. To disrupt this sequence, prey have evolved a wide variety of adaptations. Some require the two opponents to be in close proximity, such as spines, armour, and toxins, while others aim to delay or avoid encounters, including strategies like delayed hatching, hiding, fleeing, and reducing activity [4–7].

To prevent detection or attack, prey need to discriminate among a variety of external stimuli, identifying those that reliably indicate the presence and dangerousness of a predator [8]. In aquatic systems, prey can detect potential predators and assess predation risk using chemical cues [5, 9–11], including the odour of the predator itself (kairomones), alarm cues released by conspecifics, and cues from the predator’s diet. They are mostly processed through olfactory pathways [12], often involving the olfactory bulb (OB; [13], which drives the chemical information recognized by olfactory receptor neurons (ORNs) to the forebrain through mitral cells (MCs). Consistently, the ablation of MCs’ axons has been shown to cause the loss of anti-predatory responses [14]. Kairomones, consisting of a heterogeneous mixture of odorants, can activate multiple olfactory receptors, potentially inducing a complex defensive response.

Although neurophysiological studies have shown a keen interest in understanding how prey species process information to generate escape responses when exposed to predators [15], little is known about the chemical composition of predator odours and prey alarm cues. Similarly, the key neural mechanisms involved in risk perception have not been thoroughly investigated [5, 16], despite their potential to offer insights into the perception of risk itself. In behavioural experiments, the lack of a clear behavioural response is often assumed as a failure to perceive predation risk. However, this assumption may not always hold true [16]. It is crucial to recognize that the perception of risk associated with a particular stimulus can sometimes be so subtle, also depending on previous experience and life history traits, that it doesn’t trigger any significant observable reaction. This calls for a more nuanced understanding of the relationship between neurophysiological changes and behavioural responses. Evidence of such a connection has been observed in molluscs exposed to predator cues (*Lymnaea stagnalis*; [17, 18]) and mammals [19, 20]. Furthermore, embryonic exposure to predator kairomones has been shown to alter the activity of mitral cells (MCs) in *Rana dalmatina* tadpoles [21]. These findings highlight that to gain a deeper understanding of risk perception and predator-prey interactions, it is essential to relate the ecological significance of the physiological mechanisms involved in odour recognition and processing to the behavioural traits of a broad range of species [22].

Since Galvani’s experiments in the 1780s, amphibians have been widely used as animal models in various fields of research, including sensory physiology. They are easy to rear and exhibit a vast range of taxonomic diversity, which allows for comparative studies. Additionally, they are more similar to humans than many other popular animal models [23]. The anti-predatory morphological and behavioural responses of anuran larvae have been extensively investigated [24–28] and are considered a classic example of phenotypic plasticity [21, 25, 29, 30]. Tadpoles exposed to water-borne predatory cues often reduce their level of activity or incorporate unpredictable elements into their movements, increasing path complexity. The most intense responses are triggered by the synergistic effects of predator kairomones and conspecifics’ alarm cues [24, 31–34]. How exactly does the perception of cues by olfactory receptors elicit defensive responses? Based on previous research in other taxa, the perception of predation risk is thought to activate the neuroendocrine stress axis (the hypothalamus-pituitary-adrenal axis, and in amphibians, the hypothalamus-pituitary-interrenal axis), resulting in the secretion of glucocorticoids which, in turn, trigger defensive responses [15, 29].

Nevertheless, the neurophysiological changes underlying risk assessment and, consequently, the plastic response, remain largely unexplored [5, 17, 35]. It is also still debated whether scents can elicit innate responses, though it is observed that some organisms respond to predator kairomones upon first exposure [8].

To provide further evidence on how external stimuli are conveyed to the forebrain and instruct behavioural responses, we conditioned newly hatched agile frog (*Rana dalmatina*) tadpoles using kairomones (from native dragonfly larvae or non-native crayfish) either alone or coupled with conspecifics’ alarm cues. Considering that the brain undergoes modifications throughout an individual’s life depending on environmental conditions (a process known as activity-dependent neuronal plasticity; [36], we expected chronic treatments to affect the basal neuronal activity of tadpoles’ mitral cells (MCs) [37]. Long-term exposure to predation cues was expected to induce the rewiring of sensory neuronal connections and either strengthen or suppress information processing [38].

The agile frog has been shown to display strong behavioural responses to the cues of native dragonfly larvae, even in the absence of conspecific alarm cues, while non-native crayfish kairomones did not elicit any defensive response [39]. Therefore, we assumed the crayfish odour to potentially act as a neutral stimulus. Subsequently, the same neurons were acutely perfused (using the same olfactory cues of the chronic phase), and their responses assessed. We hypothesized that unconditioned tadpoles (i.e., those not previously exposed to predator cues) would provide insights into innate risk perception, and that acute cues would enhance the changes in neural parameters observed after their chronic exposure. We also expected neuronal activity to correlate with behavioural defensive responses, which were recorded in a sub-sample (ca. 60%) of previously conditioned tadpoles.

## Materials and methods

In March 2016, we collected egg mass fragments from six agile frog clutches from groundwater-fed ponds in Parco del Ticino (Bosco Castagnolo: 45°15′N, 8°58′E; Lombardy region, Northern Italy). The water depth at these sites ranged from 80 to 100 cm, with less than 10% aquatic vegetation cover. Predators, namely subadult and adult crayfish (*Procambarus clarkii*) and late instar dragonfly larvae (*Anax imperator*), were collected using dipnets (N = 12 for each predator species).

The study was conducted in accordance with current Italian laws governing amphibian collection and keeping (Prot. 0007728, 2016–2018) and the guidelines of the Italian Ministry of Health (D.M. no 68/97-A, permanent validity, issued to the Physiology Lab, Department of Biology and Biotechnology, University of Pavia). All applicable international, national, and/or institutional guidelines for the care and use of animals were adhered to.

**Fig 1:**
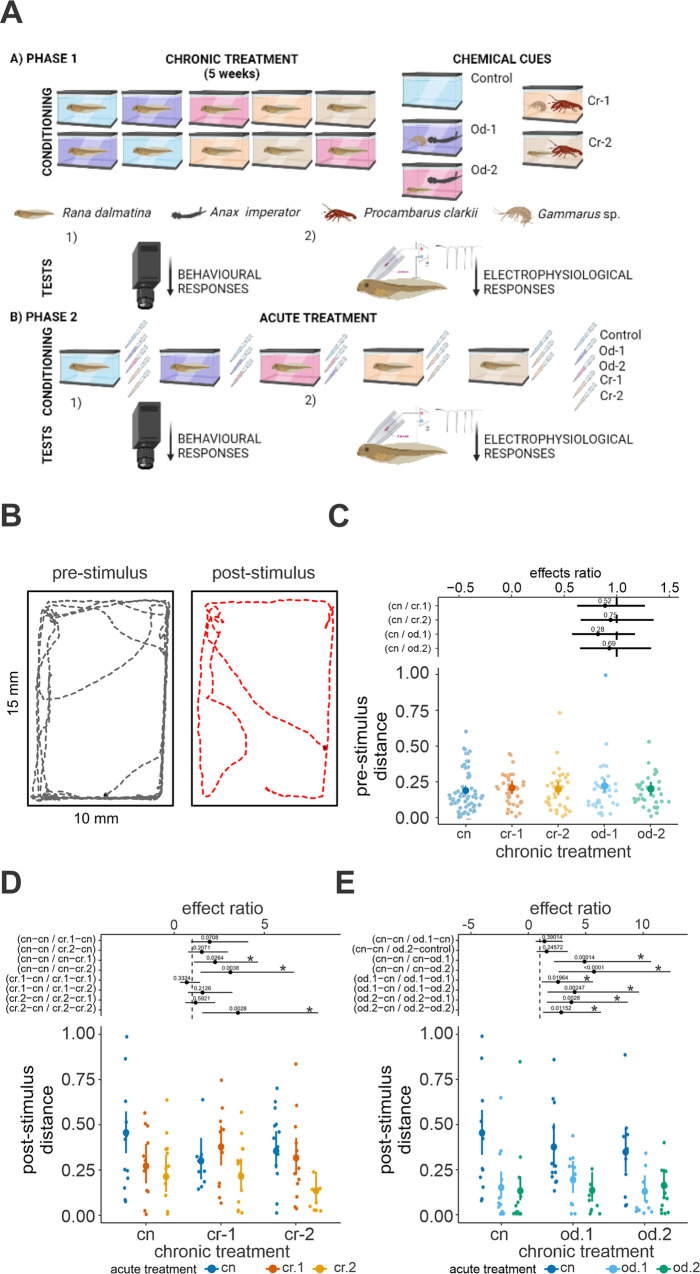
A) Diagram of the experimental design. B) Example of tadpole movement during the behavioural test before (pre-) and after (post-) stimulus injection. C) Beta GLMM estimates of the total distance travelled by tadpoles during the pre-stimulus period (n = 198). D, E) Beta GLMMs estimates of the post-stimulus total distance (D: crayfish; E: odonate). Means and 95% confidence intervals are reported as large points and bars, respectively. Treatments are compared to controls as effect ratios and their estimates and confidence intervals are reported as black points and horizontal bars, respectively. Numbers above black points are the p-values estimated for the difference of the ratio with respect to the unity (dashed vertical line). Chronic treatments are always reported before acute treatments (e.g.: cr.2-cr.1 indicates a group which received cr.2 as chronic treatment and cr.1 as acute olfactory cue). Significant differences (p < 0.05) among treatments are indicated by * and by non-overlapping effects estimates with the vertical dashed lines.

The experiment was conducted in two phases. During the first phase (the conditioning phase), tadpoles were exposed to various chemical cues for a period of five weeks. In the second phase (the testing phase), we investigated the defensive behaviour of the tadpoles and recorded their neuronal activity (Fig 1A).

### Conditioning phase

A few days after hatching (Gosner stage 25–26), two tadpoles from each clutch were placed into individual plastic containers (30 × 20 × 20 cm) filled with 8 L of aged tap water and provided with rabbit food (dry grass pellet) ad libitum. To mimic natural conditions and provide shelter, 5 g of dried leaves were added to each container. The containers were also equipped with a removable fiberglass net to facilitate the bi-weekly water change procedure with minimal disturbance to the tadpoles [6]. We arranged the containers in three spatial blocks within the laboratory, each containing 10 containers with two replicates per chemical treatment. Treatments were randomly assigned within each block, resulting in a total of 360 tadpoles (72 per treatment). The treatments were as follows: (i) 100 ml of dechlorinated water (control group, cn), (ii) 100 ml of gammarid-fed crayfish cues (cr.1), (iii) 100 ml of tadpole-fed crayfish cues (cr.2), (iv) 100 ml of gammarid-fed odonate cues (od.1), and (v) 100 ml of tadpole-fed odonate cues (od.2). Over the course of five weeks (about half of the larval development period, the length of which partly depends on environmental conditions), these treatments were gently administered to each container every morning (9–10 a.m.) for five consecutive days, followed by a two-day pause (also see [39]. The laboratory conditions were regulated to match external light and temperature conditions as closely as possible, with windows left open and temperatures maintained between 10°C and 14°C. Random checks confirmed that temperature differences among containers were ≤ 1°C.

### Olfactory cues preparation

To prepare the olfactory cues, all predators were individually housed in 0.8 L plastic tubs, each containing 0.5 L of dechlorinated water. Six predators of both types, odonate larvae and crayfish, were fed either gammarids or tadpoles (with a total weight of approximately 150–200 mg for each predator) every evening between 7 and 8 p.m. Following previous studies (e.g., [24, 31, 40]), predators were provided with live prey to mimic natural condition and obtain reliable olfactory cues. The following day, 200 ml aliquots of water were collected from each predator’s tub and then pooled in separate containers according to the predator species and diet. This concentration of cues was assumed to elicit a defensive response in tadpoles, based on findings from previous studies [6, 39, 41]. If predators did not consume their prey, they were excluded from that day’s cue preparation. The water in the predator tubs was regularly topped up to maintain a constant volume throughout the experiment.

### Effects of long-term larval conditioning on tadpole defensive behaviour during acute exposure of olfactory cues

Two days (48 hours) after concluding the previous phase, we recorded the behavioural responses of tadpoles to the five different chemical cues they had received during the conditioning phase. Tadpoles from a specific chronic olfactory treatment group were acutely exposed to either water (control cue) or both cues from the same predator species (cr.1 and cr.2 or od.1 and od.2). Tadpoles from the chronic control group were exposed to all five types of cues (Table 1). Each tadpole was exposed to only one cue type and was excluded from further experiments.

**Table 1:** Sample size (number of tadpoles tested) for each combination of chronic and acute treatments.

Chronic treatment	Acute treatment				
	control	Cr.1	Cr.2	Od.1	Od.2
**Control**	14	11	11	13	13
**Cr.1**	11	13	13	0	0
**Cr.2**	13	13	9	0	0
**Od.1**	12	0	0	14	11
**Od.2**	12	0	0	11	14

Behavioural tests were conducted over a 4-day period, aiming for 14 replicates for each combination. However, due to logistical and time constraints, the actual number of replicates varied between 9 and 14 (Table 1). To evaluate their activity before and after cue infusion, individual tadpoles were placed in tubs (15 × 10 × 10 cm), each containing 250 ml of dechlorinated water, and allowed 15 minutes for acclimatization. The tubs were arranged in a grid of six experimental units, each visually isolated from the others by cardboard barriers. An additional external barrier was used to minimize disturbance during cue injection. Each trial consisted of a 5-minute pre-stimulus period (before infusion), a 1-minute infusion period, and a 5-minute post-stimulus period (after infusion).

All trials were conducted indoors, with tadpole behaviour recorded throughout each trial using a digital video camera (Sony CCD colour video camera (PAL)) (Fig 1A and 1B). The camera was positioned above to ensure a clear and uninterrupted view of the tadpoles’ movements. To administer the chemical cues, each tub was equipped with a flexible pipe tube submerged below the water level and secured to the side of the tub. This arrangement allowed for discreet cue injection without being noticed by the tadpoles and minimized mechanical disturbance, as verified by preliminary tests. While we assumed that the tadpoles’ developmental stage did not significantly influence the direction of their response, it’s noteworthy that the intensity of responses has been reported to vary in some anuran species [30, 40]. Tadpole movements were analysed using video-tracking software (Smart v3.0, PANLAB). The total distance travelled by the tested tadpoles was used as an index of their activity level. According to our hypothesis, this activity level was expected to decrease in response to higher perceived predation risks [21].

### Testing phase: In vivo electrophysiological (patch-clamp) recordings

Tadpoles were anesthetized by dipping them for a few minutes in a small tub filled with cold water and surrounded by ice. Rapid cooling allows a more rapid anaesthesia and longer persistence time than anaesthetics commonly used for amphibians [42]. Following [21], The skin of the head was then carefully cut, and the brain was exposed along the midline for recording. Throughout the trial, both heart pulse frequency and breathing rhythm were constantly monitored. The brain was then viewed using Nomarski optics (Olympus BX51WI, Shinjuku, Tokyo, Japan). During in vivo patch-clamp recordings, tadpoles were constantly perfused, using a multibarrel perfusion system, with a fresh bath solution (pH = 7.3, 255–260 Osm) containing (mmol l^−1^): 135 NaCl, 2 KCl, 3 CaCl_2_, 1.5 MgCl_2_, 10 glucose, 10 Hepes (Sigma/Fluka, Milan, Italy). The rate of flow of the solution was regulated by a peristaltic pump. All experiments were conducted at room temperature.

Initially, we recorded the tadpoles’ baseline neural activity to assess the lingering effects of chronic treatment on their behaviour. Then, to simulate an encounter with a predator (acute exposure), tadpoles were exposed to both cues of the same predator species they had experienced during chronic exposure (cr.1 and cr.2, or od.1 and od.2; control tadpoles from chronic treatment received all four cues). Water solution with predatory cues, collected using the same procedure employed for postnatal behavioural trials, were initially dissolved in a bath solution (10 mmol l^−1^ stock) at room temperature. To exert a localized effect on the olfactory receptor neurons and prevent potential spill-over next to the recorded mitral cells, the solution was delivered in front of the olfactory pit and a rapid perfusion system was positioned near the puffing pipette, facilitating the swift removal of puffed cues. The delivery of short pressure pulses (2.7 kPa, 1.5 s) was accomplished using a Picospritzer (PDES-2L, NPI Electronic Instruments, Tamm, Germany), ejecting a small and localized quantity of solution.

To avoid dilution, neural responses were recorded 3–4 minutes after perfusing the cue into the bath solution. Before testing a different cue, we ensured a return to baseline conditions by perfusing standard extracellular solutions for at least 5 minutes. If any residual cue remained from the previous experiment, those recordings were excluded from the analysis. Each recording session lasted approximately 17 minutes.

MCs were identified based on their distinctive electrophysiological parameters, including the resting membrane potential (Vm), membrane resistance (Rm), and firing pattern, which significantly differ from those of granule cells (GCs). This differentiation is crucial for accurately targeting MCs for recordings. The olfactory bulb (OB) was patched using the blind patch approach, a technique involving electrode insertion into the MC layer without direct visual guidance [43–45]. Patch electrodes, with a tip diameter of 1–2 μm and a resistance of approximately 7–10 MΩ, were fabricated from borosilicate glass (outer diameter 1.8 mm, Hilgenberg, Malsfeld, Germany) using a two-stage electrode puller (Narishige, Tokyo, Japan). The patch pipette was filled with an intracellular solution containing (mmol l^−1^): 5 NaCl, 47 KCl, 1.5 MgCl_2_, 120 potassium gluconate, 20 Hepes, 1 EGTA, 2 Na_2_-ATP and 0.3 Na_2_-GTP (Sigma/Fluka, Milan, Italy) [44], providing the necessary ionic environment for recording MC activity.

Spontaneous excitatory postsynaptic currents (sEPSCs) were digitally filtered at 1.5 kHz, a frequency appropriate for capturing relevant synaptic events, and analysed off-line with pCLAMP10.6 (Axon Instruments) to determine peak amplitude and decay time constants. Spontaneous EPSCs were recorded in voltage-clamp mode at a holding potential of −70 mV. In contrast, cell firing activity and baseline resting membrane potential (RMP) were recorded in current-clamp mode, with 4–5 measurements per cell taken at regular intervals during the recording. Time constants for both the decay of spontaneous postsynaptic potentials (τ_EPSC_) and passive properties (τ_m_) were calculated between the 90% to 10% and 10% to 90% levels of the amplitude of each event, respectively. To assess impedance in the whole-cell configuration, voltage pulses were delivered from a microcontroller to a D/A converter and then to the patch-clamp amplifier. Series resistance was monitored by measuring passive current transients induced by −10 mV hyperpolarizing voltage steps from a holding potential of −70 mV [44–46]. We accepted deviations in transient currents of less than 15% for this parameter. Data were digitized off-line using an 8-pole Bessel filter, which helps to reduce high-frequency noise, and an A/D converter.

A 200B amplifier (Axon Instruments, Biberach an der Riss, Germany) interfaced with pClamp command/record software through a Digidata 1440A analog/digital converter (low-pass filter 10 kHz, sampling rate 100 kHz; Molecular Devices, Biberach an der Riss, Germany). These settings, including the low-pass filter and high sampling rate, were selected to optimize the fidelity and resolution of the synaptic event recordings. The frequency and amplitude of sEPSCs were recorded for MCs exposed to all chronic treatments. Quantal analysis was applied to analyse the variation in amplitude distribution [47–50]. This analysis is crucial for understanding the probabilistic nature and variability of synaptic transmission. The kinetics (τ_EPSC_) of the entire sEPSC population were calculated as the average of all sEPSCs from a dataset recorded for each MC, typically spanning at least 20 seconds. This average provides insight into the overall synaptic response characteristics of the cells under study.

### Statistical analysis

To explore tadpole behavioural responses, we divided the dataset into two groups based on the predator cues encountered during chronic treatment (odonate larvae vs. crayfish), with both groups having the same control treatment (chronic exposure to water; see Table 1). This division resulted in a 3 × 3 full factorial design for each dataset, facilitating the analysis of interacting factors and mitigating model convergence issues.

We analysed post-stimulus activity levels, expressed as total distance travelled, using two generalized linear mixed models (GLMMs) with Beta distribution and logit link function (glmmTMB package, [51]). In these models, “container within block” was initially included as a random effect. Fixed effects comprised the total distance covered before stimulus injection and both chronic and acute chemical treatments, including their interaction. After verifying no improvement in model fit, “container within block” was replaced with “container”. As beta distributions require data with observations in the open range (0, 1), we transformed both the response variable and covariate, rescaling the interval [a,b] to (0, 1) by the formula $\frac{x-a}{b-a}$. Then, we calculated $y=\frac{x(N-1)+0.5}{N}$, where *N* is the total number of observations and *x* the original response variable (Smithsen and Verkuilen, 2005).

The basal (pre-stimulus) level of activity was examined using a GLMM, which included “container” as a random intercept effect and chronic treatment as a fixed effect. This model utilized a beta distribution for the response variable and a logit link function. Tadpoles that showed no movement during the pre-stimulus period (N = 10) were excluded from the analysis of post-stimulus activity.

For membrane passive properties of MCs (capacitance, membrane resistance, and time constant), we compared controls with other treatments using nonparametric bootstrap resampling (n = 5000, dabestr package, [52]). This method is a robust alternative to parametric approaches, especially when dealing with small sample sizes or non-normal distributions. Effect sizes and 95% confidence intervals were estimated using Cumming estimation plots.

Variations in cellular inward and outward currents were analysed using GLMMs, with ’cell’ as a random intercept (4–5 measurements per cell) and chronic treatment as a fixed effect. The model for inward current used a Gaussian error distribution, while for outward current, we used a gamma distribution with logarithm as link function.

Linear models (LMs) were used to explore the effects of chronic treatments on mean sEPSC frequency and amplitude of each MC. We also ran GLMMs to investigate MC firing frequency and cell membrane potential. These models included chronic and acute treatments and their interaction as fixed effects, “cell” as a random intercept, and “cue sequence” as a random slope effect. For firing frequency analysis, a log-link function and a Gaussian distribution were incorporated. Similarly, for membrane potential analysis, an identity-link function and a Gaussian distribution were used. For each model the behavioural responses (distance) to either firing frequency or membrane potential, we set a matrix of contrasts to compare: 1) the general control group vs. the control group of each other chronic treatment; 2) within each chronic treatment, acute control groups vs. predator treatments. Results are presented as estimated means, standard errors, and confidence intervals. Both homogeneity assumptions and residual distribution of the models were checked by the simulation-based approach provided by the R package Dharma [53]. Comparisons between treatments were conducted using Dunnet’s correction method (emmeans package, [54]).

## Results

### Tadpole behavioral response to predator cues: the effect of chronic and acute exposure

Tadpole pre-stimulus activity (in the absence of any olfactory stimulus) did not show any difference between controls and the other chronic treatments (*χ*^2^ = 1.27, d.f. = 4; Fig 1C). Post-stimulus activity was highly dependent on pre-stimulus activity for both models (crayfish: *χ*^*2*^ = 34.23, d.f. = 1, *P*<0.0001; odonate: *χ*^2^ = 5.87, d.f. = 1, *P* = 0.01; crayfish slope: 2.78±0.47, *z* = 5.85, *P*<0.0001; odonate slope: 1.63±0.67, *z* = 2.42, *P* = 0.01). In general, tadpole activity after stimulus injection did not change according to either the chronic treatment (crayfish: *χ*^2^ = 3.54, d.f. = 2, *P* = 0.16; odonate: *χ*^2^ = 1.48, d.f. = 2, *P* = 0.47), or its interaction with the acute treatment (crayfish: *χ*^2^ = 6.31, d.f. = 4, *P* = 0.17; odonate: *χ*^2^ = 2.92, d.f. = 4, *P* = 0.57), but was strongly affected by the acute treatment (crayfish: *χ*^2^ = 9.71, d.f. = 2, *P* = 0.008; odonate: *χ*^2^ = 23.83, d.f. = 2, *P*<0.0001).

During behavioural tests, naïve tadpoles (control chronic treatment group) exhibited a decrease in activity when exposed to any predator cue in the acute treatment phase (Fig 1D and 1E, cn). This reduction in activity was most pronounced in response to odonate cues, regardless of the predator’s diet. Notably, tadpoles showed a stronger response to crayfish fed on tadpoles (cr.2) than to those fed on gammarids (cr.1). Although the interaction between chronic and acute treatments was not statistically significant, tadpoles exposed to chronic gammarid-fed crayfish cues (cr.1) displayed no defensive responses compared to controls when exposed to acute crayfish cues (Fig 1D). Tadpoles conditioned with cr.2, however, exhibited a clear defensive response when stimulated with the same cue. Furthermore, tadpoles exposed to acute odonate cues, both od.1 and od.2, showed a strong defensive response compared to their respective chronic control groups, regardless of the type of cue received during the conditioning phase.

### Electrophysiological characterization of MCc and GCs in tadpole OB

**Fig 2:**
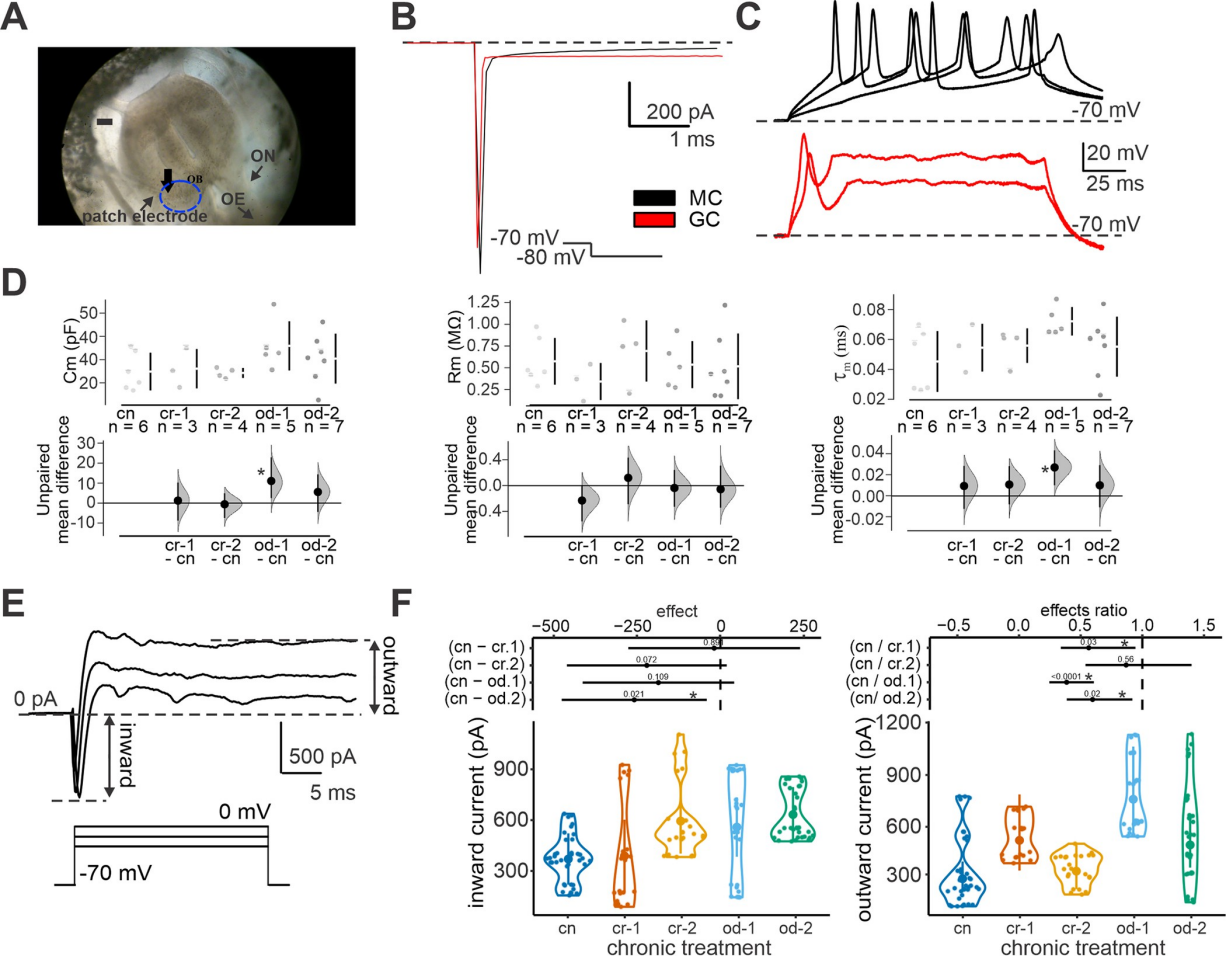
*In vivo* electrophysiological patch-clamp experimental set-up and membrane conductance repertoire analysis for MCs from control and chronically treated tadpoles. A) Typical set-up of *in vivo* tadpole’s olfactory bulb (OB, blu dashed circle) recording, with the patch-pipette *in situ* (OE: olfactory epithelium; ON: olfactory nerve). B) To analyse passive cellular properties and discriminate between mitral (MC) and granule cells (GC), transient currents were elicited by −10 mV voltage steps from a holding potential of −70 mV (see methods). C) Typical firing responses of MCs and GCs to depolarizing steps of increasing amplitude over spikes threshold. Note the delay in the occurrence of the first spike for the MC, especially with low current injections. D) Effects of chronic cue treatments on membrane passive properties (Cm = membrane capacitance, Rm = membrane resistance, (τ_m_) = membrane time constant). E) Repertoire of voltage-dependent currents elicited from a holding potential ranging from −70 mV to −10 mV in MCs from control and treated tadpoles. The interval where inward and outward current amplitudes were calculated is also shown. F) Effects of chronic treatment on inward and outward MC currents. Bottom: means and relative 95% confidence intervals estimated from GLMMs are shown as large points and thick bars within violin plots, respectively. Top: effects (mean difference or mean ratio) for all chronic treatments in comparison to controls. P-values are reported above the estimated difference/ratio of the means (black dots). Significant differences (p < 0.05) among treatments are indicated by * and by non-overlapping effects estimates with the vertical-dashed and horizontal-solid lines.

Tadpoles’ OB includes two main neuron classes: the MCs and the GCs [55, 56], which can be distinguished based on their distance from the surface of the bulb and electrophysiological properties (resting potential, input resistance and firing pattern). MCs are located in a single lamina below the GCs of the OB (Fig 2A). Compared to GCs, MCs showed significantly lower membrane resistance (319.8±22.4 MΩ, *N* = 6 vs. 168.5±6.8 MΩ, *N* = 20; *P* = 0.002; Fig 2B), and a different firing pattern, with action potentials (APs) evoked by over-threshold current injections occurring with a longer delay (54.5±9.6 ms, *N* = 5 vs. 376.0±56.3 ms, *N* = 20; *P* = 0.002) (Fig 2C). Additionally, MCs had a significantly less depolarized resting membrane potential (-52.6±3.2 mV, *N* = 6 vs. -67±4.3 mV, *N* = 20; *P* = 0.003), which may affect their overall excitability and firing dynamics.

### Effects of long-term exposure to predator cues on MC passive parameters and voltage dependent-active current

MC membrane capacitance (Cm) showed a weak tendency to increase for odonate chronic treatments in comparison to controls, with tadpoles exposed to gammarid-fed odonate cues (od.1) showing the only significant difference (S1 Table and Fig 2D). MC membrane resistance (Rm) was slightly lower for the gammarid-fed crayfish treatment, but no significant difference emerged. The membrane time constant (τ_m_) was higher for tadpoles exposed to gammarid-fed odonate cues (S1 Table and Fig 2D).

As regards neuronal active properties, depolarizing voltage steps from −70 mV to −10 mV induced in MCs a fast sodium current (inward current) and a non-inactivating outward current (Fig 2E). The inward current showed a tendency to increase across all treatments, differing significantly from controls only in tadpoles exposed to tadpole-fed odonate cues (Fig 2F). The outward current, indicative of potassium channel activity, also displayed higher values for all treatments except cr.2. Intriguingly, diets based on gammarids induced higher outward currents than those based on tadpoles in both predator types (Fig 2F), suggesting a nuanced influence of predator diet on MC activity.

### Effects of long-term tadpole exposure to predator cues on MC spontaneous activity

**Fig 3:**
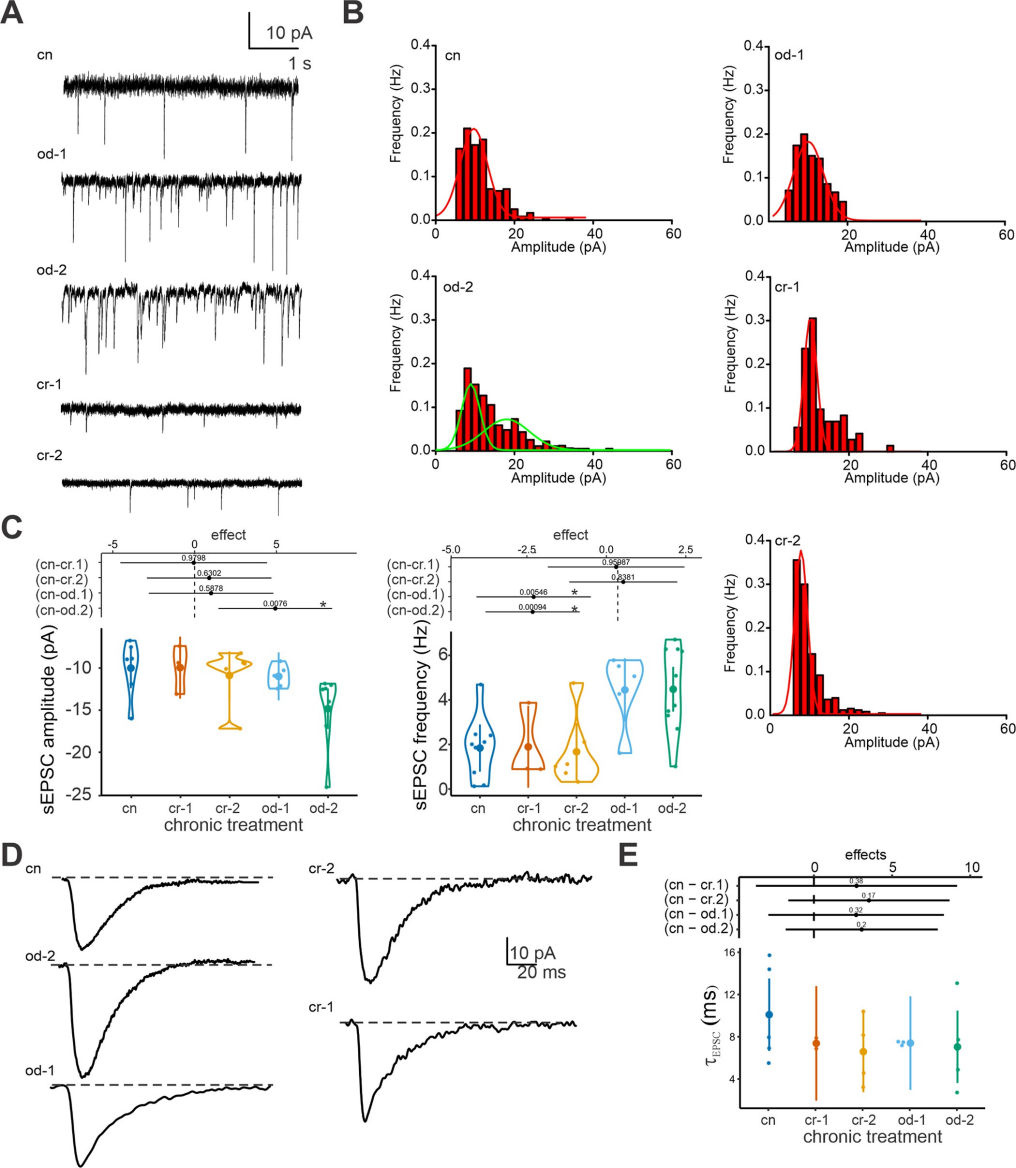
Spontaneous excitatory postsynaptic current (sEPSC) modulation after chronic exposure to cues. A) Example traces for each chronic treatment. B) Frequency distribution of sEPSCs binned at intervals of 2 pA in control and treated MCs; a multi-peak fitting was applied to od-2. C) Mean sEPSC amplitude (left) and frequency (right) of MCs from control and treated tadpoles. D) Representative sEPSCs (averaged by 10 single sEPSCs) of MCs from different chronic treatments. E) Effects of chronic treatments on membrane time constant (τ_EPSC_). Significant differences (p < 0.05) among treatments are indicated by * and by non-overlapping effects estimates with the vertical dashed lines.

The amplitude distribution of sEPSCs, binned at intervals of 2 pA, generally conformed to a single Gaussian distribution, peaking at similar values across all treatments. However, an exception was noted in the od.2 group, which exhibited a bimodal distribution (Fig 3A and 3B). This bimodal pattern in the od.2 group suggests a more complex synaptic response, potentially indicative of varied synaptic inputs or receptor types being activated. Consistent with the Gaussian distribution observed in other groups, od.2 was the only group that demonstrated a significantly higher sEPSC amplitude (Fig 3C). Additionally, both odonate treatments resulted in increased sEPSC frequency, implying enhanced synaptic activity in response to these specific predator cues.

Regarding the kinetic analysis of the postsynaptic sEPSCs, no significant variation was observed in the decay time constant (τ_EPSC_, Fig 3E), indicating that the fundamental properties of synaptic transmission remain stable.

### Effects of chronic and acute exposure to olfactory cues on the resting membrane potential and firing rate of MCs

**Fig 4:**
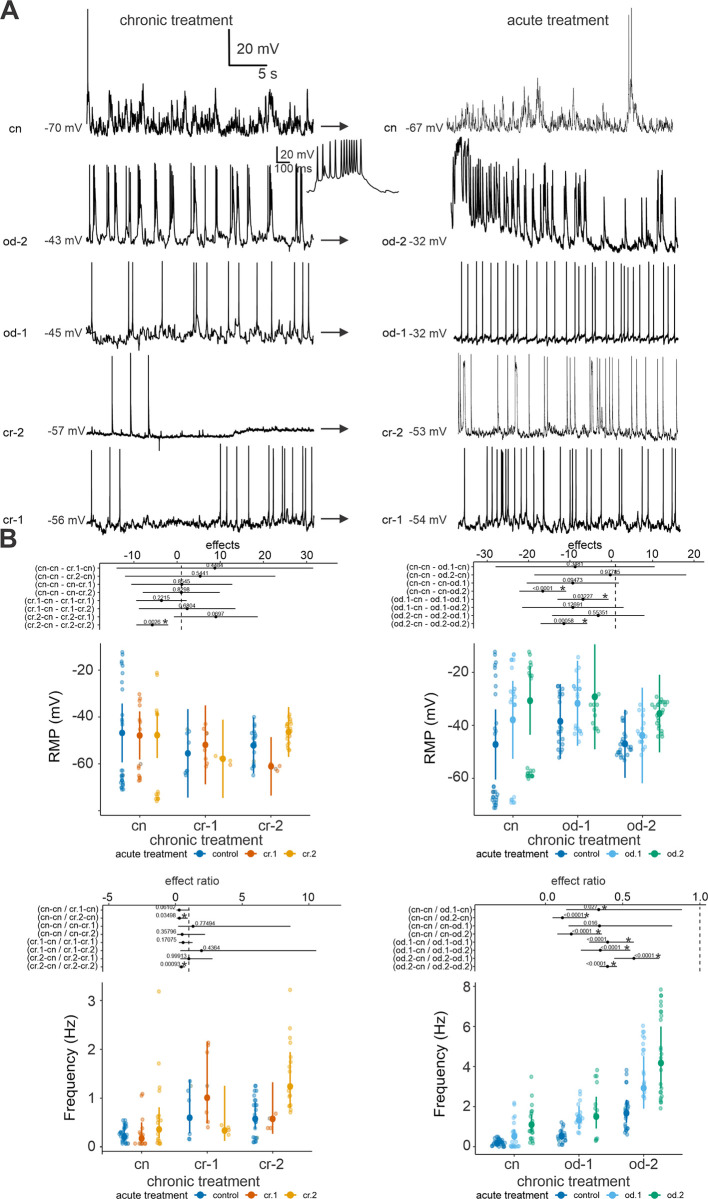
Neuronal firing modulation in different experimental conditions. A left) Representative current clamp baseline traces recorded for the different chronic treatments at the resting potential of the cells. Note the regular firing pattern in cr-1 and cr-2 vs. the bursting firing in od-2. A right) Spontaneous firing activity during the acute perfusion of the same cue used for chronic conditioning. Note the variation in RMP (mV) after acute cue perfusion. B top) GLMMs estimates for RMPs (crayfish: left; odonate: right). B bottom) GLMMs estimates of firing frequency (crayfish: left; odonate: right). Means and 95% confidence intervals are reported as large colored points and bars, respectively; for both responses, treatments are compared with controls as effect ratios and their estimates and confidence intervals are shown as black points and horizontal bars, respectively. Numbers above black points are the p-values estimated for the difference of the ratio from the unity (dashed vertical lines). Chronic treatments are always reported before acute treatment (e.g.: cr.2-cr.1 indicates a group which received cr.2 as chronic treatment and cr.1 as acute olfactory cue). Significant differences (p < 0.05) among treatments are indicated by * and by non-overlapping effects estimates with the vertical dashed lines.

Chronic larval exposure to olfactory cues did not alter the RMP of MCs (Fig 4B, dark blue bars of the two upper panels). However, a significant interaction between chronic and acute treatments was observed in the crayfish model (*χ*^*2*^ = 12.00, d.f. = 4, *P* = 0.01), indicating that the combined effects of these exposures influence MC responsiveness. In the odonate model the interaction was not significant (*χ*^*2*^ = 6.29, d.f. = 4, *P* = 0.18, see Fig 4B), showing consistent differences within each chronic treatment group. The acute treatment markedly influenced the response of MCs (*χ*^*2*^ = 237.6, 2 *df*, *P* < 0.0001), while chronic exposure had a less pronounced effect (*χ*^*2*^ = 1.23, 2 *df*, *P* = 0.53). Both acute odonate treatments induced significantly higher depolarization respect to controls (within the same chronic treatment), with od.2 showing the most relevant effect (S2 Table).

Chronic larval exposure to olfactory cues increased the baseline firing activity of MCs for all treatments (S3 Table and Fig 4B, dark blue bars of the lower panels), except for the marginally non-significant difference of the group conditioned with cr.1. This general increase in firing activity suggests heightened neural responsiveness following olfactory stimulation. In the crayfish model a significant interaction between chronic and acute treatments (*χ*^*2*^ = 15.42, d.f. = 4, *P* = 0.003) was found. Similarly, a significant interaction was noted in the odonate model (*χ*^*2*^ = 19.63, d.f. = 4, *P*<0.001). For both crayfish-conditioned groups, a significantly higher firing frequency than controls was only observed when tadpoles received the same cue in both chronic and acute exposure. For odonate groups, MC firing frequency increased compared to controls with both od.1 or od.2, and this difference was even more pronounced for tadpoles previously exposed to odonate cues; acute od.2 consistently elicited a stronger increase in firing frequency than od.1 (Fig 4B). Notably, odonate cues induced higher firing rates than crayfish cues, with the highest rates observed in the group that received od.2 during the conditioning phase (Fig 4B). When tadpoles were exposed to tadpole-fed odonate cues, the firing pattern shifted from regular to more complex (Fig 4A), indicating a profound effect of these cues on MC firing dynamics.

## Discussion

While tadpoles exposed to predation cues for five weeks did not exhibit reduced basal behavioural activity (i.e., pre-stimulus activity), at the neuronal level, tadpoles exposed to odonate cues significantly differed in both passive and active cell properties compared to those exposed to water (controls) or crayfish cues. This discrepancy suggests that while, as expected, tadpoles behave normally when no immediate threat occurs, preventing the costs of unprovoked defensive responses, chronic exposure may have altered (enhanced) their sensitivity to risk-related cues.

In response to repeated relevant stimuli, neurons can modulate both their passive (e.g., capacitance) and active (e.g., voltage-dependent conductance) properties and rewire their connections accordingly, a process thought to underlie learning and memory [57] which has been reported for several areas of the brain [29, 37, 57, 58].

The total membrane capacitance of a cell is directly proportional to the surface area and dielectric properties of the membrane [58] and plays a crucial role in the integration of electrical inputs and propagation of action potentials [59]. An increase in membrane capacitance can be determined by the growth of dendritic branches or the release of neurotransmitters from synaptic terminals via exocytosis [60]. Further experiments, correlating the passive properties of these neurons with their morphological changes are needed for disentangling the mechanisms causing the recorded increase in the membrane capacitance of tadpoles exposed to odonate cues.

The modulation of membrane conductance–that is of both the threshold and action potentials—in response to external stimuli controls neuron excitability [61]. Depolarized membrane resting potentials elicit the onset of the action potential: the early influx into the neuron of Na^+^ produces a transient inward current and a sustained outward current (delayed efflux of K^+^; [62]). In this regard, the recorded upregulation of membrane conductance can be expected to increase the responsiveness of tadpoles when exposed to the risk of being preyed on by odonate larvae.

Information on external cues (sensation) is first generated by olfactory receptor neurons but propagates along the neuronal circuit through chemical synapses. The strength of these connections can be modulated in response to relevant experiences. For this reason, we quantified both the frequency and amplitude of spontaneous excitatory postsynaptic currents (sEPSCs). Spontaneous EPSCs are synaptic inputs, produced by the opening of Na^+^ and K^+^ channels, that depolarize the postsynaptic cell, bringing the membrane potential closer to the threshold and to fire an action potential. Fast EPSPs allow the rapid transfer and transformation of coded information between the elements of the neuronal circuit. The higher the heterogeneity of the molecules composing the cue, the larger the number of receptor neurons that can be activated and consequently the frequency and amplitude of sEPSCs.

The recorded variation in the amplitude distribution of the sEPSCs of tadpoles exposed to tadpole-fed odonate cues, which showed a bimodal curve, suggests a structural remodeling of the presynaptic terminal contacting MCs. A higher frequency of sEPSCs with unaltered average amplitude is a consequence of an increase in the number of active synapses, while a second peak in the gaussian distribution may depend on either a structural enlargement of the presynaptic terminal or increased receptor density in the postsynaptic site of the synapse [49, 50, 63]. Further experiments, including the structural analysis of the synaptic connection, are needed to understand what kind of mechanism is at work.

The period of chronic conditioning did not affect tadpole behavioural response to acute treatments. As expected [21, 39], acute exposure to both odonate cues induced a strong and similar defensive response (decrease in distance covered), irrespectively of the chronic treatment. In contrast, crayfish were identified as a threat only when their kairomones were coupled with the full suite of predatory cues (including conspecifics information, as alarm cues or digestive cues). These results suggest that tadpole response is innate towards native predators and conspecific-borne chemical signals, while learning (i.e.: chronic exposure to tadpole-fed crayfish) played a negligible role in shaping the defensive responses of agile frog tadpole towards non-native crayfish. The defensive response toward the gammarid fed crayfish cue that was recorded for the chronic control group is more puzzling. Tadpole behaviour may be interpreted as a neophobic response elicited by an unknown chemical signal. However, this hypothesis is not supported by the recorded neuronal activity, which, being similar for all acute treatments, did not match tadpole behaviour. As suggested by some previous studies, crayfish odour may have been perceived as a nutrient source, rather than a potential threat [22, 39].

As for chronic treatments, the effects of acute exposure on the RMP and firing rate of MCs were evident for odonate cues, being the greatest when paired with conspecific alarm and diet cues. This pattern was consistent for all chronic treatments, confirming the additive effect of the two chemical sources of information, predator kairomones and alarm/diet cues. The clear neural response (increase in both RMP and firing frequency) observed in tadpoles exposed to tadpole fed crayfish cue, after having been conditioned with the same stimulus, demonstrates that predator alone can also affect MCs properties.

The recorded shift towards a more depolarized resting membrane potential facilitates the overcoming of the AP threshold, that is the induction of an action potential. The complex pattern of firing recorded for tadpoles exposed to both odonate stimuli may be the result of a re-arrangement in the repertoire of the voltage-dependent conductance expressed in MC membrane, which shapes the firing of the neuron [61, 64]. We suggest that, as for other brain areas, the transition to a burst firing modality may enhance the transmission of the information by recruiting a larger number of post-synaptic neurons [65].

Additionally, sustained neuronal activity and consequently calcium influx is linked to the production of reactive oxygen species (ROS), that are responsible for the increase in transcription factors needed for neuronal plasticity. We speculate that this is the cellular substrate for the long-term neurophysiological changes which drive anti-predatory behaviour [66, 67].

## Supporting information

S1 Table Means and 95% confidence intervals for MC passive proprieties (Cm = membrane capacitance, Rm = membrane resistance, τ_m_ = membrane time constant). Estimates have been obtained by non-parametric bootstrap resampling (n = 5000). Significant difference is reported in bold.
(DOCX)

S2 Table Estimated means, standard errors (SE) and 95% confidence intervals (CI) and number of MCs tested for membrane resting potential. Values were obtained from GLMMs with Gaussian error distribution using *emmeans* function (R package “emmeans”).
(DOCX)

S3 Table Estimated means, standard errors (SE), 95% confidence intervals (CI), and number of MCs tested for cell firing frequency. Values were obtained from GLMMs with log link function and Gaussian error distribution using *emmeans* function (R package “emmeans”).
(DOCX)
