# Supplementary material for: Predation cues induce predator specific changes in olfactory neurons encoding defensive responses in agile frog tadpoles
Source: PLoS One. 2024 May 2;19(5):e0302728. doi: 10.1371/journal.pone.0302728 (PMC11065311; doi:10.1371/journal.pone.0302728)
Supplement: S1 Table — Estimates have been obtained by non-parametric bootstrap resampling (n = 5000). Significant difference is reported in bold. (DOCX) [file pone.0302728.s001.docx]

| **Chronic Treatments** | **Cm (pF)** | **Rm (Mohm)** | **τ_m_ (ms)** |
| --- | --- | --- | --- |
| Cr.1 – control | 1.25 [-8.7; 10.3] | -0.23 [-0.55; 0.01] | 0.01 [-0.01; 0.02] |
| Cr.2 – control | -0.59 [-7.4; 4.9] | 0.12 [-0.29; 0.43] | 0.01 [-0.01; 0.03] |
| **Od.1 – control** | **11.10 [2.5; 22.9]** | **-0.04 [-0.32; 0.24]** | **0.03 [0.01; 0.04]** |
| Od.2 – control | 5.57 [-4.4; 14.4] | -0.06 [-0.34; 0.31] | 0.01 [-0.01; 0.03] |

ST.1. Means and 95% confidence intervals for MC passive proprieties (Cm = membrane capacitance, Rm = membrane resistance, τ_m_ = membrane time constant). Estimates have been obtained by non-parametric bootstrap resampling (n = 5000). Significant difference is reported in bold.
