# Supplementary material for: Predation cues induce predator specific changes in olfactory neurons encoding defensive responses in agile frog tadpoles
Source: PLoS One. 2024 May 2;19(5):e0302728. doi: 10.1371/journal.pone.0302728 (PMC11065311; doi:10.1371/journal.pone.0302728)
Supplement: S2 Table — Values were obtained from GLMMs with Gaussian error distribution using emmeans function (R package “emmeans”). (DOCX) [file pone.0302728.s002.docx]

| **Chronic treatment** | **Acute treatment** | **Estimated mean** | **SE** | **df** | **Cells (n)** | **95% CI** |
| --- | --- | --- | --- | --- | --- | --- |
| Control | Control | -46 | 5 | 115 | 6 | -56 – (-35) |
| Cr.1 | Control | -55 | 9 | 115 | 2 | -73 – (-37) |
| Cr.2 | Control | -52 | 6 | 115 | 5 | -64 – (-40) |
| Control | Cr.1 | -50 | 5 | 115 | 5 | -61 – (-40) |
| Cr.1 | Cr.1 | -51 | 9 | 115 | 2 | -70 – (-34) |
| Cr.2 | Cr.1 | -53 | 6 | 115 | 1 | -66 – (-41) |
| Control | Cr.2 | -50 | 5 | 115 | 4 | -61 – (-40) |
| Cr.1 | Cr.2 | -60 | 9 | 115 | 1 | -78 – (-41) |
| Cr.2 | Cr.2 | -46 | 6 | 115 | 5 | -57 – (-34) |
| Control | Control | -45 | 5 | 179 | 6 | -55 – (-34) |
| Od.1 | Control | -38 | 6 | 179 | 5 | -50 – (-27) |
| Od.2 | Control | -47 | 5 | 179 | 6 | -57 – (-36) |
| Control | Od.1 | -40 | 5 | 179 | 5 | -50 – (-29) |
| Od.1 | Od.1 | -31 | 6 | 179 | 5 | -43 – (-20) |
| Od.2 | Od.1 | -44 | 5 | 179 | 4 | -54 – (-33) |
| Control | Od.2 | -31 | 5 | 179 | 5 | -41 – (-20) |
| Od.1 | Od.2 | -28 | 6 | 179 | 3 | -40 – (-16) |
| Od.2 | Od.2 | -35 | 5 | 179 | 6 | -46 – (-25) |

ST.2. Estimated means, standard errors (SE) and 95% confidence intervals (CI) and number of MCs tested for membrane resting potential. Values were obtained from GLMMs with Gaussian error distribution using *emmeans* function (R package “emmeans”).
