# Supplementary material for: Predation cues induce predator specific changes in olfactory neurons encoding defensive responses in agile frog tadpoles
Source: PLoS One. 2024 May 2;19(5):e0302728. doi: 10.1371/journal.pone.0302728 (PMC11065311; doi:10.1371/journal.pone.0302728)
Supplement: S3 Table — Values were obtained from GLMMs with log link function and Gaussian error distribution using emmeans function (R package “emmeans”). (DOCX) [file pone.0302728.s003.docx]

| **Chronic treatment** | **Acute treatment** | **Estimated mean** | **SE** | **df** | **Cells (n)** | **95% CI** |
| --- | --- | --- | --- | --- | --- | --- |
| Control | Control | 0.11 | 0.06 | 117 | 6 | 0.04 - 0.31 |
| Cr.1 | Control | 0.57 | 0.25 | 117 | 2 | 0.24 -1.37 |
| Cr.2 | Control | 0.50 | 0.13 | 117 | 6 | 0.29 – 0.84 |
| Control | Cr.1 | 0.21 | 0.08 | 117 | 5 | 0.09 – 0.47 |
| Cr.1 | Cr.1 | 0.94 | 0.40 | 117 | 2 | 0.40 – 2.20 |
| Cr.2 | Cr.1 | 0.59 | 0.17 | 117 | 1 | 0.33 – 1.05 |
| Control | Cr.2 | 0.39 | 0.14 | 117 | 4 | 0.19 – 0.78 |
| Cr.1 | Cr.2 | 0.27 | 0.15 | 117 | 1 | 0.09 – 0.80 |
| Cr.2 | Cr.2 | 1.17 | 0.28 | 117 | 5 | 0.72 – 1.89 |
| Control | Control | 0.17 | 0.08 | 184 | 6 | 0.07 – 0.42 |
| Od.1 | Control | 0.49 | 0.13 | 184 | 5 | 0.29 – 0.83 |
| Od.2 | Control | 1.60 | 0.29 | 184 | 6 | 1.11 – 2.31 |
| Control | Od.1 | 0.53 | 0.14 | 184 | 5 | 0.32 – 0.89 |
| Od.1 | Od.1 | 1.28 | 0.27 | 184 | 5 | 0.84 – 1.96 |
| Od.2 | Od.1 | 3.08 | 0.56 | 184 | 4 | 2.15 – 4.41 |
| Control | Od.2 | 1.09 | 0.23 | 184 | 5 | 0.71 – 1.67 |
| Od.1 | Od.2 | 1.62 | 0.34 | 184 | 3 | 1.06 – 2.48 |
| Od.2 | Od.2 | 4.16 | 0.75 | 184 | 6 | 2.91 – 5.94 |

ST.3. Estimated means, standard errors (SE), 95% confidence intervals (CI), and number of MCs tested for cell firing frequency. Values were obtained from GLMMs with log link function and Gaussian error distribution using *emmeans* function (R package “emmeans”).
